# The use of *Lactobacillus plantarum* 299v (DSM 9843) in cancer patients receiving home enteral nutrition – study protocol for a randomized, double-blind, and placebo-controlled trial

**DOI:** 10.1186/s12937-020-00598-w

**Published:** 2020-09-11

**Authors:** Karolina Kaźmierczak-Siedlecka, Marcin Folwarski, Karolina Skonieczna-Żydecka, Jakub Ruszkowski, Wojciech Makarewicz

**Affiliations:** 1 Department of Surgical Oncology, Medical University of Gdansk, Mariana Smoluchowskiego 17, 80-214 Gdansk, Poland; 2 Department of Clinical Nutrition and Dietetics, Medical University of Gdansk, Dębinki 7, 80-211 Gdansk, Poland; 3 Department of Human Nutrition and Metabolomics, Pomeranian Medical University in Szczecin, Broniewskiego 24, 71-460 Szczecin, Poland; 4 Department of Physiopathology, Medical University of Gdansk, Dębinki 7, 80-211 Gdansk, Poland; 5 Department of Nephrology, Transplantology and Internal Medicine, Medical University of Gdansk, Dębinki 7, 80-211 Gdansk, Poland

**Keywords:** *Lactobacillus plantarum* 299v, Home enteral nutrition, Cancer, Nutritional status

## Abstract

**Background:**

Nutritional treatment is one of the most important components of multidisciplinary anti-cancer therapy. Home enteral nutrition is considered as a safe procedure, however, it may be associated with the risk of side effects, such as nausea, vomiting, abdominal pain, and diarrhoea. It is uncertain whether diarrhoea is the result of the enteral formula administration or gut dysbiosis. One of the methods which may be used to alter the composition of gut microbiota is the administration of a probiotic strain. *Lactobacillus plantarum* 299v ingestion was found to diminish the adverse events of irritable bowel syndrome and *Clostridium difficile* infection - entities that share the symptoms with enteral nutrition side effects. Therefore, the primary aim of this study is to determine the effect of *Lactobacillus plantarum* 299v on prevention of weight loss of cancer patients receiving home enteral nutrition. The secondary aims are to evaluate the role of this probiotic strain in the improvement of nutritional status, enteral nutrition tolerance, and patients’ quality of life.

**Methods:**

Forty patients with cancer receiving home enteral nutrition will be enrolled in this clinical trial and randomized to receive one capsule of *Lactobacillus plantarum* 299v (Sanprobi IBS®) twice a day or placebo for 12 weeks in a double-blind manner. Laboratory tests (the level of albumin, total protein, transferrin, and total lymphocyte count), anthropometric parameters (body mass, the content of fat mass, muscle mass, and total body water), Nutritional Risk Screening (NRS 2002), enteral nutrition tolerance as well as quality of life will be measured. Measurements will be obtained at the baseline and after 4 and 12 weeks of treatment.

**Discussion:**

The adverse events observed during administration of enteral nutrition have an negative impact on enteral formula tolerance and as a consequence patients’ quality of life. The previous studies have demonstrated that probiotics may reduce the gastrointestinal symptoms related to enteral nutrition. Thus, administration of *Lactobacillus plantarum* 299v may be effective in improvement of nutritional status, enteral nutrition tolerance, and quality of life of cancer patients receiving home enteral nutrition.

**Trial registration:**

ClinicalTrials.gov Identifier: NCT03940768.

## Background

Nutritional treatment is essential within the complexity of anti-cancer therapy. The appropriate nutritional support improves nutritional status, clinical outcomes, and as a consequence patients’ quality of life [1]. Home enteral nutrition (HEN) is recommended for patients with an efficiently functioning gastrointestinal tract who do not require hospital stay [2]. According to the Villar Taibo et al. trial, almost 75% of patients qualified for HEN are malnourished [3]. Moreover, it is estimated that around half of patients with malignancies eventually develop cancer cachexia [4, 5]. As it was stated above, HEN provides many benefits, however, it is associated with the risk of adverse events, mainly diarrhoea, nausea, vomiting as well as abdominal pain [2]. The frequency of diarrhoea varies, notwithstanding, it is estimated that it affects as much as 95% of patients receiving enteral nutrition (EN) depending on medical condition and the definition of diarrhoea being a multifactorial etiology [6]. Therefore, it should be considered whether diarrhoea is the side effect of EN (for instance – it is mainly caused by the unadjusted speed of the enteral formula administration) or it is caused by alterations in the composition as well as the activity of gut microbiota. The qualitative and quantitative changes in gut microbiota commonly referred as gut dysbiosis may occur in cancer patients [7]. It was elegantly shown that there are at least several factors contributing to the development of gut microecological niche imbalance among patients suffering from malignancies, mainly infectious and anti-cancer agents, antibiotics as well as poor eating habits [7, 8].

Currently, several therapeutic methods are used to alter the gut microbiota, predominantly administration of prebiotics, probiotics, synbiotics, and faecal microbiota transplantation [9, 10]. Probiotic strain – *Lactobacillus plantarum* 299v (*L. plantarum* 299v) is able to reside the human colonic mucosa in vivo due to a specific mechanism of mannose adhesion [11, 12]. It demonstrates high tolerance to acidic and alkaline environment of the stomach and the duodenum, respectively. Therefore, this probiotic strain survives transit through the gastrointestinal tract to the colon, where it can modify gut microbiota [8, 11, 12]. *L. plantarum* 299v provides anti-bacterial activity against potential pathogenic agents, such as *Listeria monocytogenes*, *Yersinia enterolytica*, *Enterobacter cloacae*, *Enterococcus faecalis* and *Escherichia coli*. It increases transcription of the genes encoding mucins (MUC2 and MUC3) and their secretion from goblet cells [13]. *L. plantarum* 299v has immunomodulatory properties reducing the pro-inflammatory cytokine synthesis and increasing the anti-inflammatory IL-10 production and secretion. This probiotic strain plays a supportive role in the treatment of irritable bowel syndrome (IBS), which was confirmed in Niedzielin et al. trial [14]. The participants were divided into two groups: first receiving probiotic (*n* = 20) in dose 200 ml (5 × 10^7^ CFU/ml) twice a day for 4 weeks and second consuming placebo (n = 20). All the participants from probiotic group declared a resolution of abdominal pain, while only 11 subjects from control group experienced relief (*p* = 0.0012). The normalization of stool frequency was most commonly observed in probiotic group in comparison to placebo (*p* = 0.17). Moreover, the improvement of all symptoms of IBS was noted in 95% subjects receiving probiotic and in 15% participants from control group (*p* < 0.0001) [14]. It should be emphasized that patients receiving HEN often develop similar gastrointestinal symptoms resembling IBS [2]. Limited data exists regarding the use of *L. plantarum* 299v in cancer therapy and no trials about its use in patients with cancer receiving HEN. The present randomized, double-blind and placebo-controlled study hypothesizes that administration of *L. plantarum* 299v might improve nutritional status, enhance the EN tolerance and quality of life (QOL) of patients with cancer who receive HEN.

## Methods

### Study aims

The primary aim of the study is to determine the effect of *L. plantarum* 299v on prevention of weight loss of patients with cancer receiving HEN. The secondary aims include the role of *Lactobacillus plantarum* 299v in the improvement of nutritional status, EN tolerance, and QOL of patients with cancer receiving HEN.

### Study design

**Fig. 1:**
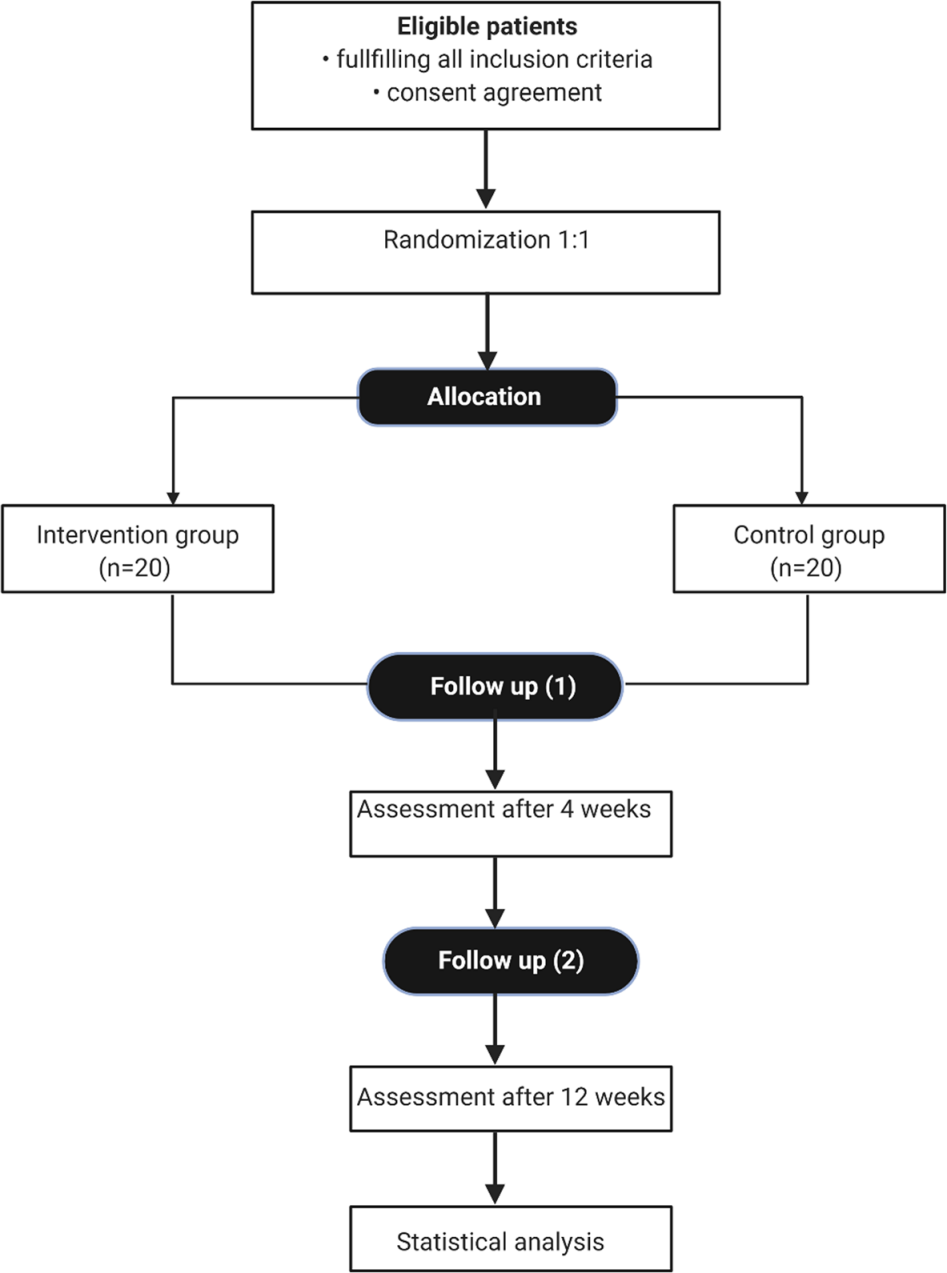
Participants flow diagram

In this 12-week, single-centre, randomized, double-blind, and placebo-controlled study, 20 patients with cancer will be treated with probiotics and 20 patients will receive a placebo. The post-allocation clinical and laboratory assessment will be performed at weeks 0, 4, and 12 in all participants (Table 1). Patients in the probiotic group will receive two capsules of Sanprobi IBS® containing 10^10^ CFU of *L. plantarum* 299v for 12 weeks. Participants in the control group will receive placebo (two capsules daily) for 12 weeks. The participants will be recruited to this study by the nutritionist and the surgeon in Nutritional Counselling Centre Copernicus in Gdansk and Medical University of Gdansk (Department of Clinical Nutrition and Dietetics). The researches and participants will be blinded to the group assignment since the probiotic product and the placebo will be completely identical and indistinguishable from one another. The enrolment of participants is presented in Fig. 1.

**Table 1:**
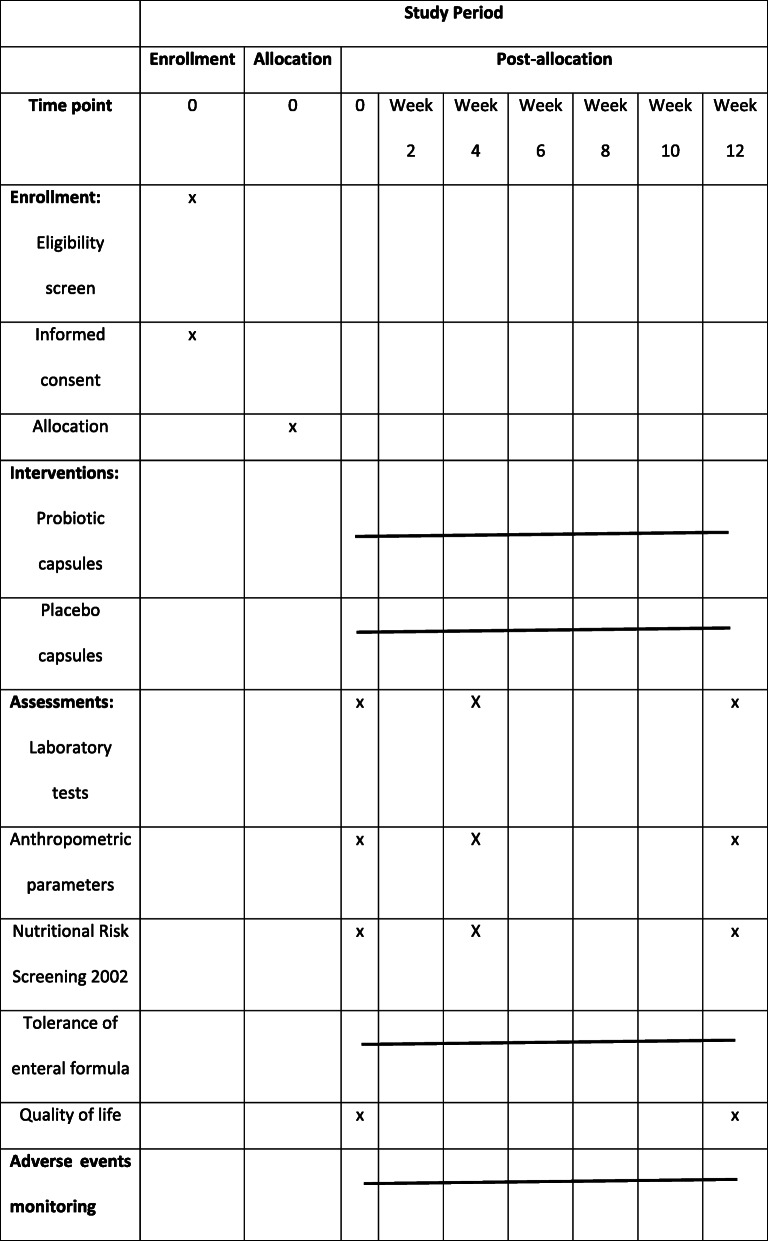
Schedule of enrollment, interventions, and assessments.

### Subjects

#### Inclusion criteria

Patients will be included if they meet all of the following criteria:

- Age ≥ 18 year
- The presence of cancer
- Artificial access to the gastrointestinal tract (naso-gastric tube, gastrostomy, percutaneous endoscopic gastrostomy, jejunostomy, percutaneous endoscopic gastrostomy with jejunostomy) – enteral feeding
- Qualification for HEN
- written consent to take part in the study

#### Exclusion criteria

Patients will be excluded if they meet any of the following exclusion criteria:

- Qualification for home enteral nutrition, but suffering from another disease than cancer
- Patients requiring additional parenteral nutrition
- Not being able to visit the study centre

### Allocation to treatment, randomization, and blinding

At the beginning, the patients will have to sign an informed consent to participate in the study. After meeting all the inclusion criteria and obtaining a consent agreement, the participants will receive a unique number by the nutritionist. Each number will be allocated to particular intervention group. The eligible patients will be allocated to the treatment for 12 weeks with either to probiotic group or placebo.

Randomization will be performed by means of randomizer.com software, typically used by clinical research associates. The randomization ratio will be 1:1. The researches and participants will be blinded for treatment received from the probiotic company. The patients will intake one capsule of study product (probiotic or placebo) in the morning after breakfast and one capsule in the evening after dinner. The participants who cannot swallow the capsule, will be instructed to mix the capsules’ powder with 20 ml of water or saline and administer the solution through the enteral feeding access. The capsules (probiotic product as well as placebo) will be stored at refrigerator temperature.

#### Intervention group

The intervention group will be administered with Sanprobi IBS® capsule twice a day for 12 weeks. Each capsule contains 10^10^ CFU of *L. plantarum* 299v. The intervention capsules will be produced and packed by Sanprobi company (Sanprobi IBS® Sanprobi Sp. z o.o., Sp. k., Szczecin, Poland; producer of capsules – Institute Rosell-Lallemand, Montreal, Canada; LP299v owner of probiotic strain – Probi AB, Lund, Sweden).

#### Control group

The control group will receive placebo capsules twice a day for 12 weeks. The placebo capsules will be produced and packed by the same company (Sanprobi) as intervention capsules and it will not contain any microorganisms. One capsule of placebo 410 mg +/− 7.5% of contents, including potato starch – 403 mg and 7 mg of magnesium stearate (magnesium salts of fatty acids).

### Outcomes

#### Primary outcome

The primary outcome is the prevention of weight loss in a probiotic-receiving group in comparison to a placebo-receiving group.

#### Secondary outcomes

First secondary outcome is the improvement of nutrition status in patients receiving probiotic in comparison to placebo-receiving group. The nutritional status will be evaluated by means of anthropometric parameters (body mass index, fat mass, muscle mass, total body water), laboratory tests (the level of albumin, total protein, transferrin, total lymphocyte count) and Nutritional Risk Screening 2002 (NRS 2002 tool). Second secondary outcome is to estimate the differences of enteral nutrition tolerance in patients receiving placebo or probiotic supplements. Third secondary outcome is to evaluate the difference of QOL of patients receiving placebo or probiotic supplements.

### Data collection

#### Participant timeline

The timetable of follow-up visits and measurements is presented using The Standard Protocol Items: Recommendations for Interventional Trials (SPIRIT) diagram in the Table 1. Each of the participants will visit the study centre three times (at baseline, after 4 weeks, and after 12 weeks). During follow-up visits, patients will receive study products depending on allocation. At baseline – 60 capsules for 30 days; after 4 weeks – 120 capsules for next 60 days. If during this trial, any participant fails to continue the study protocol, the data will be collected and this information will be further noted in the publication.

#### Blood sampling and preparation

The level of albumin, total protein, transferrin, and total lymphocyte count will be measured. The blood samples will be taken from a forearm vein by a nurse at the baseline, after 4 weeks, and next after 12 weeks. All blood samples will be taken in Counselling Nutritional Centre Copernicus in Gdansk and the same day they will be given to the laboratory to conduct the analysis.

#### Anthropometric parameters

The analysis of the body mass composition (fat mass, muscle mass, total body water) will be conducted using a BIA analyser – Medical Jawon. It will be performed by a nutritionist in the Department of Clinical Nutrition and Dietetics (at baseline, after 4 weeks, and after 12 weeks). The body mass index will be also calculated by the nutritionist.

#### NRS 2002 tool

This is a validated screening tool divided into two parts. The first one assesses the nutritional status based on unintentional weight loss during last 1 to 3 months and food intake during last 1 to 3 weeks. The second part regards the occurrence of diseases or types of treatment which are related to increased daily calories intake (e.g. bone marrow transplant, radio- or chemotherapy). Patients aged 70 and above receive additional score. Nutritional support should be provided if the NRS score is 3 and more. Assessment of nutritional status will be conducted by a nutritionist in the Department of Clinical Nutrition and Dietetics using NRS 2002 tool at baseline, after 4 weeks, and after 12 weeks.

#### EN tolerance

The EN tolerance will be evaluated by the nutritionist in the Department of Clinical Nutrition and Dietetics using own questionnaire including information, such as number of stools, occurrence of nausea, vomiting, abdominal pain, and flatulence. The questionnaire will be filled every day for 12 weeks by patients or their caregivers and evaluated during follow-up visits after 4 and 12 weeks of the study.

#### Quality of life

Patients’ QOL will be assessed by the nutritionist in the Department of Clinical Nutrition and Dietetics. To assess the patients’ QOL the World Health Organization Quality of Life-BREF (WHOQOL-BREF) questionnaire will be used. It contains questions divided into 4 domains (environmental, psychological, somatic, and social factors). The QOL will be evaluated at baseline, after 4 and 12 weeks.

### Adverse outcomes

Patients are instructed to inform the researchers about any changing conditions during the trial. Furthermore, during follow-up visits the researchers will ask about adverse events which may be related to the intervention. If any adverse events occur it will be noted in report form and reported in publication. However, according to the best of our knowledge, adverse events after administration of *L. plantarum* 299v were not noted.

### Ethical approval

The study protocol has been approved by the Independent Bioethics Committee for Scientific Research at Medical University of Gdansk (the project indentification code: 422/2016). All participants will give informed consent before randomization and the information about the trial will be explained to them. They will be informed about potential benefits and adverse events that may occur during this trial.

### Clinical trial registration

The study has been registered in ClinicalTrials.gov which is a database of privately and publicly funded clinical studies conducted around the world (ClinicalTrials.gov Identifier: NCT03940768).

### Statistical analysis

When computing a priori sample size, we assummed that of all existing approaches used in screening and assessment of malnutrition and cachexia, weight loss – among others - is a phenotype always evaluated by international organizations and commonly used tools/surveys [15], that this parameter will serve as pivotal to calculate required sample size. Calculating the mean weight loss (major component of nutritional status) in a 70 kg men and assuming 1:1 allocation ratio and 80% statistical power we evaluated that the number of participants will be 36. Allowing for a withdrawal rate of 20% for the primary outcome we aim to randomly allocate 40 participants to receive either active product or placebo. The required sample size were evaluated using G-power analysis software. Significance level will be 0.05.

The calculations will be carried out with the use of Statistica package by Dell Inc. The descriptive statistics will include averages, medians, standard deviations, maximum and minimum values. In order to check the normality of distribution of populations subject to research, the W Shapiro-Wilk test will be applied. To check the homogeneity of variations of the groups compared, the Brown-Forsythe test will be applied. Then, depending on the type of data and shape of distributions compared, the U Mann-Whitney test, Student’s t-test or a version of Student’s t-test with independent variance estimation will be applied.

## Discussion

To the best of our current knowledge, this is the first randomized, double-blind, and placebo-controlled trial assessing the role of *L. plantarum* 299v in the prevention of weight loss, improvement of nutritional status, EN tolerance, and QOL of cancer patients receiving HEN.

The beneficial effects of *L. plantarum* 299v for several diseases have been demonstrated in many clinical trials [14, 16]. Hoppe et al. reported that administration of *L. plantarum* 299v increases iron absorption in women [17]. Additionally, as it was stated above, *L. plantarum* 299v is effective in supporting treatment of IBS. Moreover, in Dudzicz et al. study, it was confirmed that routine use of *L. plantarum* 299v may prevent *Clostridium difficile* infection (CDI) during antibiotic therapy in patients receiving immunosuppressing agents and being hospitalized in nephrology and transplantation ward [18]. Furthermore, the results of Wullt et al. study, indicated that *L. plantarum* 299v reduces the side effects of antibiotics on colonic fermentation in patients suffering from recurrent *Clostridium difficile*-associated diarrhoea [19]. The significant reduction of antibiotic-associated gastrointestinal symptoms (the frequency of loose stools and nausea) after administration of *L. plantarum* 299v has been confirmed in a double-blind and placebo-controlled Lönnermark et al. trial [20].

EN provides many benefits for cancer patients, mainly preventing mucosal atrophy, reducing endotoxins translocation, and preserving gut immunity; however, as it was stated above, it is associated with adverse events, while the most common one is diarrhoea [2, 21]. The incidence of diarrhoea is increased in critically ill patients [22]. Probiotic strains may be used to prevent side effects of EN which was shown in Zhao et al. study [23]. This trial recruited 120 patients with gastric cancer receiving EN in combination with probiotics for 7 days after the surgical procedure. The patients were divided into 3 groups: first received fiber-free nutrition formula (*n* = 40), second consumed fiber-enriched nutrition formula (n = 40), and third received fiber- and probiotics-enriched nutrition formula (n = 40). Patients receiving probiotics-enriched nutrition formula had a lower risk of developing diarrhoea. However, the laboratory parameters assessing nutritional status did not differ significantly between groups [20]. The similar results were obtained by Xie et al. [24]. In this study including 140 patients with gastric cancer receiving EN and probiotics for 8 days, the decrease in the incidence of diarrhoea and reduction of the pro-inflammatory cytokines level (IL-6, IL-8, and TNF-α) in the postoperative period were reported. However, the laboratory parameters assessing nutritional status did not change [22]. Overall, the results of these above mentioned studies confirm that probiotic strains are effective in reducing the occurrence of diarrhoea. Notwithstanding, due to short period of administration of EN and probiotics (7 and 8 days), it was difficult or almost impossible to improve the nutritional status of cancer patients.

In our study, we decided to administer the probiotic strain *L. plantarum* 299v in full dose (2 × 10^10^ CFU) for 12 weeks, which allows us to expect changes in anthropometric and laboratory parameters (especially in cases of parameters with a long half-life, for instance in case of albumin). The possibility of *L. plantarum* 299v to reduction of gastrointestinal symptoms related to EN may contribute to improvement of EN tolerance and as a consequence to improve patients’ QOL.

There may be some possible limitations in the design of the study:

- First, this is a single centre study with small sample size; moreover, the group is non-homogenous. It could take a lot of time to recruit more patients and with the same type of cancer, due to high mortality of cancer patients qualified for HEN. Additionally, not every patients fulfilling inclusion criteria is able to take part in this study. Therefore, there is a need to create a multi-center study to have a better chance for a larger sample size.
- Second, the participants will intake the capsules at home, so we cannot be completely certain if they will really administer the intervention products.
- Third, the tolerance of enteral nutrition will be assessed by questionnaire fulfilled by patients at home, thus we cannot be sure if they will do it every day or simply before follow-up visits.

## Data Availability

It is only a study protocol, so the data are not available yet.

## Abbreviations

HEN: home enteral nutrition
EN: enteral nutrition
*L. plantarum* 299v: *Lactobacillus plantarum* 299v
OQL: quality of life
NRS 2002: Nutritional Risk Screening 2002 tool
WHOQOL-BREF: World Health Organization Quality of Life-BREF
CDI: *Clostridium difficile* infection
